# Respiratory Disease Related Mortality and Morbidity on an Island of Greece Exposed to Perlite and Bentonite Mining Dust

**DOI:** 10.3390/ijerph10104982

**Published:** 2013-10-14

**Authors:** Stefanos Sampatakakis, Athena Linos, Eleni Papadimitriou, Athanasios Petralias, Archontoula Dalma, Eirini Saranti Papasaranti, Eleni Christoforidou, Melina Stoltidis

**Affiliations:** 1 Hellenic Center for Disease Control and Prevention (HCDCP), 3-5 Agrafon street, Athens 15235, Greece; 2 Department of Hygiene, Epidemiology and Medical Statistics, Medical School, National and Kapodistrian University of Athens, 75 Mikras Asias street, Athens 11527, Greece; E-Mails: a.linos@prolepsis.gr (A.L.); e.papad@yahoo.gr (E.P.); a.petralias@prolepsis.gr (A.P.); n.dalma@prolepsis.gr (A.D.); irene.sarant@gmail.com (E.S.P.); echristof@med.uoa.gr (E.C.); mstoltidi@gmail.com (M.S.); 3 Institute of Preventive Medicine, Environmental & Occupational Health, Prolepsis, 7 Fragoklisias street, Athens 15125, Greece; 4 Department of Statistics, Athens University of Economics and Business, 76 Patision street, Athens 10434, Greece

**Keywords:** chronic obstructive pulmonary disease, respiratory, perlite, bentonite, mining dust

## Abstract

A morbidity and mortality study took place, focused on Milos Island, where perlite and bentonite mining sites are located. Official data concerning number and cause of deaths, regarding specific respiratory diseases and the total of respiratory diseases, for both Milos Island and the Cyclades Prefecture were used. Standardized Mortality Ratios (SMRs) were computed, adjusted specifically for age, gender and calendar year. Tests of linear trend were performed. By means of a predefined questionnaire, the morbidity rates of specific respiratory diseases in Milos, were compared to those of the municipality of Oinofita, an industrial region. Chi-square analysis was used and the confounding factors of age, gender and smoking were taken into account, by estimating binary logistic regression models. The SMRs for Pneumonia and Chronic Obstructive Pulmonary Disease (COPD) were found elevated for both genders, although they did not reach statistical significance. For the total of respiratory diseases, a statistically significant SMR was identified regarding the decade 1989–1998. The morbidity study revealed elevated and statistically significant Odds Ratios (ORs), associated with allergic rhinitis, pneumonia, COPD and bronchiectasis. An elevated OR was also identified for asthma. After controlling for age, gender and smoking, the ORs were statistically significant and towards the same direction.

## 1. Introduction

The adverse effect of mining dust on the respiratory status of mining site workers, especially coal miners and the workers exposed to silica and asbestos, has been up to date adequately studied, both in developed and developing countries [1,2,3]. It is a common knowledge that construction workers—especially those exposed to silica containing dust—and workers in mining sites are at an increased risk of developing pneumoconiosis [4,5]. As far as workers exposed to silica and cumulative respirable dust are concerned, a significant increase in Chronic Obstructive Pulmonary Disease (COPD) independent of smoking, has been observed [6,7]. 

International agencies concerned about the health and safety of workers, recognize as hazardous materials regarding respiratory health, apart from crystalline quartz and asbestos, other non-fibrous mineral dusts as well, which are classified as “nuisance” dusts, such as kaoline, bentonite and perlite. These may increase the risk for the exposed individuals to develop lung cancer and mesothelioma [8].

Perlite is an amorphous volcanic glass, composed of SiO_2_ (71%–75%), Al_2_O_3_ (12%–16%), Na_2_O (3%–4%), K_2_O (4%–5%), Fe_2_O_3_ (0.5%–2%), MgO (0.2%–0.7%), CaO (0.5%–1.5%) and 2%–5% bound water. It originates from volcanic eruptions and it is typically formed by the hydration of obsidian. In order to be used for industrial purposes, perlite is heated at temperatures up to 1,000 degree Celsius, in order to become what is known as expanded perlite, which is characterized by low density, high surface area and low thermal conductivity. It is mainly used as a soil conditioner, packaging material, construction insulator for walls and floors, agriculture substrate and as a filter aid. Perlite is regarded as a generally safe material, and the time weighted average of threshold limit value (TLV-TWA) is 10 mg/m^3^ without short-term exposure limit (American Conference of Government Industrial Hygienists) [9]. According to the International Federation of Chemical, Energy and General Workers’ Unions, the threshold limit value for perlite is 5 mg/m^3^ [10].

Up to date, little literature evidence exists regarding perlite exposure effects on workers’ and populations’ morbidity—especially regarding their respiratory health and pulmonary function—and mortality. More specifically, according to Cooper [11], who conducted a study on workers occupied in perlite mining and processing factories and as a result, were exposed to perlite dust for up to 23 years, it was found that their exposure in perlite dust was around the official limits, and that there was no evidence of pneumoconiosis in 152 perlite workers by using chest radiography or pulmonary function tests [12]. On the other hand, about thirty years ago, an orchid grower exposed to perlite, developed pulmonary irritation and mild hemorrhage, as perlite crystals were found in the upper bronchi [13].

There are only two recent studies examining the effect of perlite dust on the respiratory health of exposed workers available. The first [14], took place in Turkey, where pulmonary function tests and chest radiograms were performed in 36 perlite exposed workers and 22 unexposed office workers in the same perlite industry plant twice, with an interval of four years. Respirable perlite dust levels exceeded the official limits in the processing area. In this study, the authors reached to the conclusion that although 12-year perlite exposure did not lead to decreased spirometric indices, a four-year decline in Transfer Function of the Lung for Carbon Monoxide (T_L_CO) was significant in perlite workers, which indicates a possible small airway obstruction. In the second study [15], followed 24 workers exposed to perlite in an industry located in Taiwan for 6 months, after an accidental explosion of a nitrogen tank, whose insulator consisted mainly of expanded perlite powder. The symptoms observed among the workers within the first 6 months were cough, eye irritation, shortness of breath and throat irritation. Moreover, three of them, during this period of time, showed obstruction of the PFT (Pulmonary Function Tests) and specifically predicted Forced Expiratory Volume in one second (FEV_1_) <80%. These three cases fulfill the term of Reactive Airway Dysfunction Syndrome (RADS).

Bentonite is a clay mineral, which contents silica in percentages which range from less than 1% to more than 20%. Up to date, several studies have shown that exposure—especially occupational—to silica, may be associated mainly with lung and kidney malignancies in workers [16,17]. Moreover, according to International Agency for Research on Cancer [18], the carcinogenicity of crystalline silica depends on the “inherent characteristics of the material”. Bentonite also contains mica, feldspar along with other minerals in different proportions. The use of bentonite is widely spread in chemical, pharmaceutical, cosmetic and food industry, civil, geotechnical and environmental engineering, agriculture and mining. Humans are exposed to bentonite through occupational and environmental pathways, mostly by the respiratory tract and dermal contact.

Up to date, few studies have described pulmonary fibrosis after workers’ occupational exposure to bentonite dust [19,20]. Examining the potential mechanisms by which such a relationship may occur, a recent study, where the cytotoxic effect of bentonite particles was examined *in vitro* on human lung fibroblasts, it was found that the cytotoxic potential of bentonite may strongly be associated with the fibroblasts membrane lysis [21]. Furthermore, researchers found that bentonite particles may induce the cytotoxicity and oxidative stress, as well as genotoxicity in human lymphoblast B cells [22,23].

Milos (Cyclades, Greece) is an Aegean island, where perlite and bentonite mining sites are primarily located, and as a result, is one of the leading worldwide mining sites regarding these materials. We hypothesized that continuous exposure to perlite and bentonite dust would adversely affect Milos’ permanent residents’ respiratory health. Therefore, in order to examine the potential effects of elevated exposure to these materials contained in the suspended mining dust, we performed an epidemiological morbidity and mortality study in Milos Island. It should be noted that this is the first study where this type of association is examined, as well as the only study where a population of this size is permanently residing in an area where exposure in perlite and bentonite dust takes place.

## 2. Experimental Section

### 2.1. Study Area Location and Exposure

**Figure 1:**
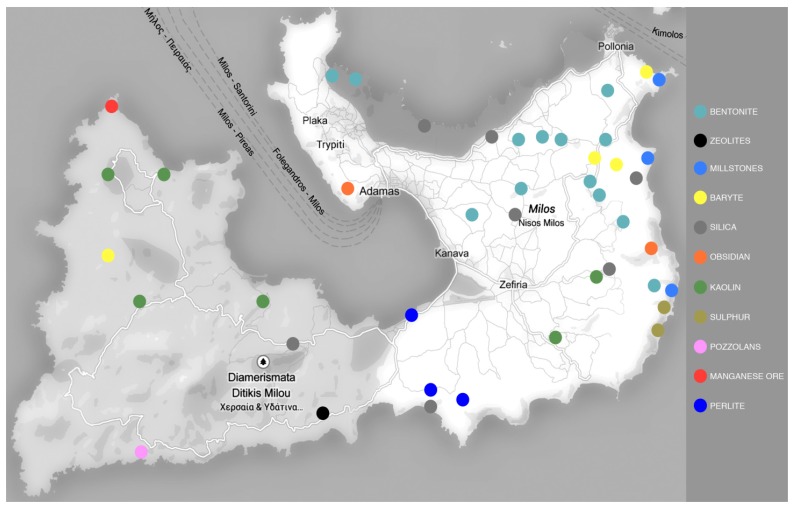
Historical and modern mining locations of mineral products in Milos. Current production includes mining locations of bentonite and perlite.

The mortality study was conducted in the island of Milos, which is located 140 km southeast of Athens. It is the 5th largest island of the Cyclades Prefecture (which includes 24 islands in the Aegean). The fact that makes Milos special, compared to the rest of the islands that form the Cyclades Prefecture, is that Milos is a product of volcanic activity which started some 2–3 million years ago and stopped 90,000 years ago. There are two inactive volcanos on the island. As a product of volcanic activity, the island of Milos is rich in natural mineral resources. The most significant ones can be listed as follows: bentonite, perlite, pozzolana and small quantities of caolin. One of the primary factors of Milos’ economic growth is the mining of these mineral resources. Due to fact that perlite and bentonite have mined at Milos Island since the late 1950s, Greece has evolved into a leading country worldwide in the production of these minerals, producing yearly more than 1.15 million metric tons of perlite (crude and processed), with a maximum of 1.75 million metric tons in 2007, and more than 1.6 million metric tons of bentonite (crude and processed), with a maximum of 2.84 million metric tons in 2008 (yearly statistics of the Greek Mining Enterprises Association). There are more than 100 mining sites located in the island, 25 of which are active perlite and bentonite mines [24]. Most of them are found in the north-eastern part of the island, which constitutes Milos’ main residential area (Figure 1). According to official land cover data of the Hellenic Statistical Authority, in 2000, 95.8% of the island of Milos was covered by shrubs, little or no vegetation associations and agricultural areas, while only 0.95% of the island was covered by urban fabric. However, mining sites covered a significant 3.2% of the island surface (5.4 out of 167.7 thousand acres). Note in this, that all these mines correspond to large surface sites and not underground sites.

The island of Milos, like the rest of the Cyclades Prefecture, experiences specific climatic conditions that refer to strong northern winds during July and August, also known as “Cycladic Meltemia”. Furthermore, in Milos, wind intensity is estimated to be rather high, i.e., more than 6 B (Beaufort) for at least a quarter of the year (Hellenic National Meteorological Service data). The particular climatic facts, combined to the wide surface covered by the perlite and bentonite mining sites, are likely to affect residents’ exposure in perlite and bentonite dust. 

The morbidity study was conducted in the island of Milos, along with the municipality of Oinofita. The municipality of Oinofita is located 50 km north of Athens, in the Voiotia Prefecture. The Oinofita area has evolved into an industrial region since the late 1970s and has seen increased scientific interest [25], because of the industrial waste produced by the large number of industries operating in the area (around 700 according to [26]). According to the registry of industries held by the Oinofita municipal office, the industrial zone of Oinofita hosts numerous types of industrial activity, such as chemical production, detergents, pesticides and pharmaceuticals, leather production, aluminium, food production and fodders.

It is clear that the two areas under study have significant differences, with respect to their geographical terrain and the risk factors that affect public health. In the island of Milos, the basic risk factor is associated with the mining dust, while in the Oinofita area with the air, ground and water pollution mainly caused by industrial waste. Although on one hand this factor increases the complexity of the study, on the other hand, it makes the analysis more interesting, mainly due to the fact that we have a chance to compare populations exposed to different risk factors, which both affect respiratory health and pulmonary function. In order to scan for potential differences in the characteristics of the two populations, the confounding factors of gender, age and smoking have been taken into account in the analysis.

### 2.2. Study Population

For the mortality study, the data used were provided by the Hellenic Statistical Authority. That data concerned the number and cause of deaths, stratified by gender, age (divided in 5 year groups) and calendar year, for both the island of Milos and the Cyclades prefecture. The authorities provided data for specific respiratory diseases (ICD9 range: 460–519) and lung cancer (ICD9 162) related deaths for the 11-year period 1999–2009, while for the total of respiratory diseases (ICD9 range: 460–519) related deaths, the provided data was for the 21-year period 1989–2009.

For the morbidity study, a predefined questionnaire was used, and a face to face interview took place for 269 persons residing in the island of Milos (with a total population of 4,771 people according to the 2001 census) during April 2011 and April 2012, along with 1,811 persons in the municipality of Oinofita (with a total population of 8,195 according to the 2001 census) during the time period between January 2010 and April 2011. The criteria of eligibility for participating in the study were that the participants were permanent residents, thus exposed to the aforementioned health risk factors in both study areas. No exclusion criteria were used regarding age, gender or health status. For children under 15 years of age, the interviews were taken from their mothers.

### 2.3. Questionnaire

The questionnaire employed, included demographic and socioeconomic characteristics, such as gender, age, place of birth, nationality, educational status, marital status, number of children and siblings and employment status. The questionnaire also included specific questions on participants’ smoking habits, since smoking is the main confounding factor associated with respiratory disorders. The main part of the questionnaire was associated with participants’ health status. More specifically, participants were asked if they have ever been diagnosed by a medical doctor with specific respiratory system diseases (ICD9 range: 460–519), as well as lung cancer (ICD9 162); each disease was asked separately.

### 2.4. Statistical Analysis

In order to study the mortality due to respiratory diseases in the island of Milos, we calculated person years and observed deaths, stratified by gender, age (divided in 5 year groups), and calendar year. The expected number of deaths was calculated on the basis of the Cyclades Prefecture, to which the island of Milos belongs to. All related data for the mortality study were provided by the Hellenic Statistical Authority, thus the classification system used by the authority [27] is employed to classify the respiratory diseases. Standardized Mortality Ratios (SMRs) were computed, adjusted to age (in 5-year age groups), gender and calendar year, by dividing the observed number of deaths with the expected number of deaths (multiplied by 100). Confidence intervals of 95% and *p*-values were calculated for the SMRs on the basis of the exact Poisson method [28,29]. Tests of linear trend were performed, after computing SMRs (adjusted for age and gender) for each year of follow up.

For the morbidity study, we computed the percentage of people having specific respiratory diseases in the two areas (Milos and Oinofita) and performed Chi-square and Fisher exact tests. The Odds ratio (with the associated confidence interval) of Milos with respect to Oinofita was calculated for each disease. In order to account for the confounding factors of gender, age and smoking, we performed binary logistic regression for each respiratory disease, controlling for these factors. The statistical analysis was performed using the statistical software SPSS 17 (IBM, Athens, Greece).

## 3. Results and Discussion

### 3.1. Results

#### 3.1.1. Mortality

The observed deaths, expected deaths, SMR’s and corresponding *p*-values and confidence intervals comparing the island of Milos to the Cyclades Prefecture are presented in Table 1. In terms of total number of deaths in Milos, they are lower than expected, compared to the Cyclades Prefecture, although the difference is not statistically significant. As far as specific diseases of the respiratory system are concerned, the SMRs for Pneumonia (SMR = 128) and Chronic Obstructive Pulmonary Disease (COPD) (SMR = 160) are increased for both males and females, but do not achieve statistical significance. On the other hand, the SMR associated with lung cancer, appears to be lower and does not statistically differ with respect to that of the Cyclades Prefecture. Linear trend was not identified for the specific respiratory diseases during the 11-year period 1999–2009.

**Figure 2:**
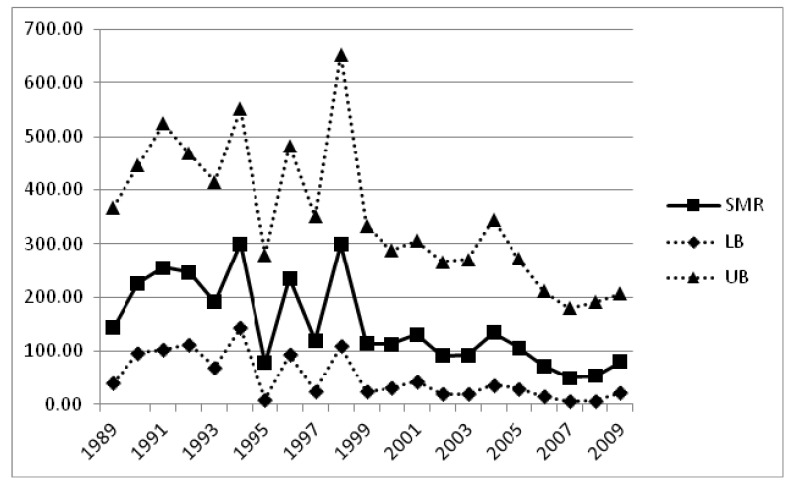
SMRs (with 95% CI) of Milos compared to Cyclades prefecture for the total of respiratory diseases (ICD9 range: 460–519); years 1989–2009.

Data provided by the statistical authority with regards to deaths from the total of respiratory diseases (ICD9 range: 460–519) for the 21-year period 1989–2009, revealed the existence of a decreasing trend (*p*-value < 0.001) (Table 2). Interestingly enough, the SMR (CI) for the period 1989–1998 is equal to 211.9 (162.4–271.5) (and statistically significant for both males and females), while for the period 1999–2009 equal to 92.1 (64.8–126.9). As depicted in Figure 2, the yearly SMRs, with the exception of year 1995, are consistently higher during the period 1989–1998, as compared to the time period between 1999 and 2009.

#### 3.1.2. Morbidity

Table 3 depicts the morbidity results in Milos as compared to Oinofita. We observe that the Odds ratios associated with allergic rhinitis (OR = 2.37), pneumonia (OR = 5.97) and COPD (OR = 2.16) are elevated and remain statistically significant. The Odds Ratio (OR = 1.58) for asthma is of borderline significance (Fischer’s *p*-value = 0.051). A significantly elevated Odds Ratio is found regarding bronchiectasis (OR = 13.56), but this is taken with caution, due to the small number of cases (two in Milos and one in Oinofita). Furthermore, it is of note that there were no reported cases of pneumonoconiosis (ICD9 500–508) or pulmonary fibrosis (ICD9 515) in either Milos or at Oinofita.

The mean age of the two populations (Milos 49.9, Oinofita 48.4) does not differ statistically (*p*-value = 0.260). In addition to that, there is no statistically significant difference with respect to gender (female are 49.4% in Milos and 46.9% in Oinofita). However, there is a statistical significant difference (*p*-value = 0.021) with respect to smoking. In Milos, the proportion of current smokers is 24.5% and that of ex-smokers equal to 29%; the other 46.5% never smoked. At Oinofita, the respective proportions are comparatively lower; that of current smokers equal to 26.3% and that of ex-smokers equal to 18.8%. After controlling for these factors via logistic regression, it is made obvious within the last rows of Table 3, that the Odds ratios of the above reported respiratory diseases are again statistically significant and towards the same direction.

**Table 1:** Mortality in Milos compared to Cyclades prefecture for specific respiratory diseases; years 1999–2009.

Disease	Total Deaths	Accute respiratory infections	Pneumonia	Chronic Obstructive pulmonary disease (COPD) and extrinsic allergic alevolitis	Pneumonoconioses	Other diseases of the respiratory system	Cancer of Lung, trachea and bronchus
**ICD9 range**		**460–462, 465**	**480–486**	**495, 496**	**500–508**	**488, 510, 512–519**	**162**
**Total**							
SMR (CI)	90.61 (83.00–98.73)	66.03 (30.19–125.35)	128.31 (41.66–299.43)	159.80 (87.36–268.11)	70.25 (1.78–391.40)	65.52 (28.29–129.10)	79.79 (51.63–117.78)
Observed deaths	521	9	5	14	1	8	25
Expected deaths	574.98	13.63	3.90	8.76	1.42	12.21	31.33
*p*-value	0.0239	0.2562	0.7020	0.1249	1.0000	0.2834	0.2954
Trend test (*p*-value)	0.9706	0.8375	0.4737	0.6626		0.2032	0.9739
SMR 1999–2002 (CI)	89.07 (76.91–102.59)	35.94 (0.91–200.27)	139.21 (16.86–502.87)	202.19 (74.20–440.09)	0.00 (0.00–1578.18)	106.09 (38.93–230.92)	102.36 (51.10–183.15)
Obs. deaths 1999–2002	192	1	2	6	0	6	11
SMR 2003–2006 (CI)	91.74 (79.12–105.79)	79.22 (21.58–202.84)	173.88 (35.86–508.15)	173.82 (56.44–405.63)	258.76 (6.55–1441.73)	24.79 (0.63–138.14)	41.92 (13.61–97.83)
Obs. deaths 2003–2006	189	4	3	5	1	1	5
SMR 2007–2009 (CI)	91.28 (76.78–107.71)	68.98 (18.79–176.62)	0.00 (0.00–502.01)	102.85 (21.21–300.56)	0.00 (0.00–459.20)	39.66 (1.00–220.96)	103.92 (47.52–197.27)
Obs. deaths 2007–2009	140	4	0	3	0	1	9
**Male**							
SMR (CI)	92.00 (81.52–103.45)	80.61 (29.58–175.46)	122.12 (25.18–356.89)	166.58 (83.16–298.06)	0.00 (0.00–414.78)	123.96 (49.84–255.40)	80.75 (49.99–123.44)
Observed deaths	279	6	3	11	0	7	21
Expected deaths	303.26	7.44	2.46	6.60	0.89	5.65	26.00
*p*-value	0.1697	0.7720	0.8900	0.1455	0.8200	0.6744	0.3805
**Female**							
SMR (CI)	89.06 (78.20–101.02)	48.49 (10.00–141.70)	138.86 (16.82–501.63)	139.04 (28.67–406.33)	187.99 (4.76–1047.40)	15.24 (0.39–84.89)	75.06 (20.45–192.19)
Observed deaths	242	3	2	3	1	1	4
Expected deaths	271.72	6.19	1.44	2.16	0.53	6.56	5.33
*p*-value	0.0726	0.2705	0.8440	0.7318	0.8251	0.0213	0.7695

**Table 2:** Mortality in Milos compared to Cyclades prefecture for the total of respiratory diseases (ICD9 range: 460–519); years 1989–2009.

	Total	Male	Female
SMR (CI)	142.55 (115.85–173.54)	150.74 (115.03–194.03)	131.54 (93.54–179.83)
Observed deaths	99	60	39
Expected deaths	69.45	39.80	29.65
*p*-value	0.0010	0.0034	0.1135
Trend test (*p*-value)	<0.0001	0.0056	0.0787
SMR 1989–1998 (CI)	211.81 (162.39–271.53)	199.65 (137.43–280.38)	227.58 (152.41–326.84)
Obs. deaths 1989–1999	62	33	29
SMR 1999–2009 (CI)	92.09 (64.84–126.93)	116.00 (76.45–168.78)	59.15 (28.37–108.79)
Obs. deaths 1999–2009	37	27	10

### 3.2. Discussion

This epidemiological study is consisted of two parts, a mortality part and a morbidity part. According to the mortality study’s results, the number of deaths during the predefined eleven-year period 1999–2009, was higher in the island of Milos regarding pneumonia and chronic obstructive pulmonary disease (COPD), in comparison to the expected number of deaths in Cyclades Prefecture for the same respiratory disorders (increased SMRs—not reaching statistical significance). The SMR for the total of respiratory diseases during the period 1989–1998 was elevated and statistically significant, whilst decreased in the following decade. We are not aware of any significant event occurring in the island (*i.e.*, changes in the active mining sites or the total production of bentonite or perlite), which could explain this trend. This decrease can be attributed to either an improvement in the mining-extraction procedures, or to an easier access to health system services.

**Table 3:** Respiratory disease related morbidity in Milos compared to Oinofita.

Disease	Allergic rhinitis	Pneumonia	Asthma	Bronchiectasis	Chronic obstructive pulmonary disease (COPD.)	Respiratory failure	Cancer of Lung, trachea and bronchus
**ICD9**	**477**	**480–486**	**493**	**494**	**496**	**518.81–518.84**	**162**
Milos% (N = 269)	5.2 (14)	5.9 (16)	9.7 (26)	0.7 (2)	4.1 (11)	0.4 (1)	0.4 (1)
Oinofita% (N = 1,811)	2.3 (41)	1.0 (19)	6.4 (115)	0.1 (1)	1.9 (35)	0.4 (8)	0.3 (5)
Chi-square test (*p*-value)	0.005	<0.001	0.044	0.006	0.025	0.870	0.785
Fischer’s exact test (*p*-value)	0.012	<0.001	0.051	0.046	0.041	1.000	0.565
							
OR (CI)	**2.37 (1.27–4.41)**	**5.97 (3.03–11.75)**	1.58 (1.01–2.47)	**13.56 (1.23–150.04)**	**2.16 (1.09–4.31)**	0.84 (0.11–6.75)	1.35 (0.16–11.58)
							
OR (CI) and *p*-value controlling for gender, age and smoking (logistic regression)	**2.24 (1.20–4.19)**	**5.47 (2.73–10.97)**	1.49 (0.95–2.34)	**12.47 (1.09–143.05)**	**2.28 (1.11–4.66)**	0.83 (0.10–6.74)	1.37 (0.16–11.99)
	**0.011**	**<0.001**	0.082	**0.043**	**0.024**	0.858	0.774

The morbidity part of the study was conducted in two industrial types of environment, the island of Milos (perlite and bentonite mining sites—ambient air polluted area) and the municipality of Oinofita (air, water and ground pollution—mostly due to industrial waste), areas with similar demographic characteristics. It was found that the prevalence of allergic rhinitis, pneumonia and COPD was higher on the island of Milos compared to the municipality of Oinofita, while a statistically significant association was observed. Similar were the results found for bronchiectasis, despite the small number of the observed cases, while regarding asthma, the difference was of borderline significance. We can therefore infer that factors related to the exposure to perlite and bentonite dust—Milos’ permanent residents—may contribute to their respiratory health related mortality and morbidity higher rates.

To this day, there are only two recent studies where a relationship between exposure to perlite dust and respiratory health status is observed. Polatli *et al.* [14] who conducted a study on perlite workers exposed to low levels of perlite for more than 10 years and concluded to a four-year decline in Transfer Function of the Lung for Carbon Monoxide (T_L_CO), which reflects a small airway obstruction. Regarding the second study, Du *et al.* [15] followed 24 workers in an industry in Taiwan for 6 months, after an acute exposure to perlite dust, due to an accident which occurred at the industry where they worked. Within the first 6 months, the workers developed respiratory tract disorders such as cough, shortness of breath and throat irritation. Moreover, three of them, during this period of time, developed signs of Reactive Airway Dysfunction Syndrome (RADS) and performed a Forced Expiratory Volume in one second (FEV_1_) less than 80%. Both studies were conducted in working places. In the first one, the long term effects of exposure to perlite dust are being studied, while in the second one, its acute effects. In our study, a whole population is under study, based on their permanent residential area and not just their working place. Finally, in agreement with our findings, Cooper *et al.* [12,13] proved no relation between long term exposure to perlite dust and pneumonoconiosis.

As far as bentonite is concerned, it should be noted that only one case of pneumonociosis because of exposure to bentonite dust (sodium montmorillonite) has been described by Gibbs and Pooley [20], while in a previous study, Phibbs *et al.* [19] described silicosis in bentonite workers. No epidemiological study in general population is up to date available regarding both exposure to perlite and bentonite mining dust.

Our findings are supported by the fact that an existing recent study concluded to the fact that bentonite particles have a cytotoxic effect on human lung fibroblasts through their membrane lysis [21]. In addition to that, a previous study performed in guinea pigs by McMichael *et al.* [30] found that their exposure to high concentration of perlite dust can cause lymphoid aggregation and perivascular inflammatory response.

Our study is limited by the fact that we did not use data concerning the perlite and bentonite dust concentration in ambient air and their potential to exceed the officially set limits. Furthermore, occupational confounding factors were not taken into account in the analysis. Moreover, whether these effects on residents’ respiratory health are due to bentonite and perlite dust separately, or if these dusts have a cumulative effect on the development of residents’ respiratory disorders remains a question that could not be answered by our study.

This is the first epidemiological study where specific methodology is used to indicate bentonite’s and perlite’s dust role in the mortality and morbidity rate of permanent residents. Moreover, this is the first study comparing a region (the island of Milos) exposed to mining dust, to an industrial residential area (the municipality of Oinofita) without such mining exposure. In the immediate future, we intend to conduct a study focused on other health disorders, such as eye irritation [15], which may be caused by exposure in perlite dust. Furthermore, our immediate intention is to conduct a similar type of morbidity study accompanied by a spirometry test performance on workers at bentonite and perlite mining sites, as well as elementary school children, in order to identify if their respiratory health may be affected by such a dust exposure.

## 4. Conclusions

People—permanent residents of Milos island—exposed to perlite and bentonite mining dust, indicate an increased risk of developing diseases of the respiratory system, such as pneumonia, chronic obstructive pulmonary disease and allergic rhinitis. The morbidity for these diseases is elevated and statistical significant, while the mortality for pneumonia and chronic obstructive pulmonary disease is elevated, although non-statistically significant. More morbidity studies with detailed exposure data are needed, in order to examine the effects of different types of mining dust to exposed populations.
